# Evaluation of the need for preoperative short hookwire placement for small pulmonary lesions

**DOI:** 10.1007/s11604-025-01807-4

**Published:** 2025-05-29

**Authors:** Noriyuki Umakoshi, Toshihiro Iguchi, Hiroyuki Ujike, Toshiharu Mitsuhashi, Yusuke Matsui, Koji Tomita, Soichiro Okamoto, Kazuaki Munetomo, Seiichiro Sugimoto, Shinichi Toyooka, Takao Hiraki

**Affiliations:** 1 Department of Radiology, Okayama University Hospital, 2-5-1 Shikata-Cho, Kitaku, Okayama 700-8558 Japan; 2 Department of Radiological Technology, Faculty of Health Sciences, Okayama University, Okayama, Japan; 3 Department of General Thoracic Surgery and Breast and Endocrinological Surgery, Dentistry and Pharmaceutical Science, Okayama University Graduate School of Medicine, Okayama, Japan; 4 Center for Innovative Clinical Medicine, Okayama University Hospital, Okayama, Japan; 5 Department of Radiology, Faculty of Medicine, Dentistry and Pharmaceutical Sciences, Okayama University, Okayama, Japan

**Keywords:** Lung, Video-assisted thoracic surgery, Pulmonary lesion, Preoperative localization, Interventional radiology

## Abstract

**Purpose:**

Although preoperative marking is often required to accurately locate the targets for video-assisted thoracic surgery, target lesions can be identified intraoperatively without marking in some cases; however, the frequency and characteristics of these lesions remain unclear. Therefore, we aimed to retrospectively evaluate the need for a short hookwire for preoperative localization of small pulmonary lesions.

**Materials and methods:**

Computed tomography (CT)-guided short hookwire placement was performed for 176 lesions (mean diameter, 7.9 ± 3.5 mm) in 171 sessions prior to video-assisted thoracoscopic surgery. Placement was performed if one or more of the following CT findings were present: lesions (1) ≤ 10 mm in diameter; (2) ≥ 5 mm from the pleural surface, and (3) predominantly consisting of ground-glass opacity. The need for a hookwire for intraoperative lesion detection was retrospectively assessed based on surgical records. Factors associated with the absence of a hookwire for lesion detection were determined using univariate and multivariate analyses.

**Results:**

Placement was successful in all cases; however, the hookwire was dislodged at the time of surgery in four lesions (2%). Among the remaining 172 lesions, thoracoscopic resection was performed using a hookwire as a landmark in 101 lesions (58.7%), whereas 71 lesions (41.3%) were detectable without a hookwire. Previous ipsilateral lung resection significantly increased the odds of not needing a hook wire (OR 4.24; *P* = 0.005). Larger target lesions (mean, 8.4 vs. 7.1 mm) and those located further from the pleura (mean, 13.3 vs. 8.0 mm) were associated with an increased need for hook wires. Multivariate analysis revealed that experienced surgeons required more hookwires compared to trainees (*P* = 0.029). Solid nodules did not require hookwires (*P* = 0.032).

**Conclusion:**

Shallow solid lesions in patients with a history of ipsilateral lung resection may not require hookwire placement during resection, even if they are small.

## Introduction

Video-assisted thoracic surgery (VATS) is widely used in the treatment of lung cancer (both primary and secondary) and offers a minimally invasive alternative to traditional open surgery. This procedure is sometimes employed to target lesions that are modest in size, located away from the pleural surface, or ground-glass nodules. Given the minimally invasive nature of this surgical approach, preoperative marking is often required to accurately locate the targets. Various marking techniques, such as dye and lipiodol injections, and mammographic needles, have been employed for this purpose [1]. Among them, the short hookwire-and-suture system (Guiding-Marker System; Hakko, Tokyo, Japan) is frequently adopted in Japan, with numerous researchers attesting to its effectiveness [1, 2].

Although preoperative marking may be performed based on the specific criteria of each institution or thoracic surgeon, the target lesion can be identified intraoperatively without marking in some cases, despite marking according to traditional criteria. This may be because of the evolution and refinement of surgical procedures, enhanced proficiency of thoracic surgeons, and advancement of the equipment used. Consequently, determining lesions that do not require preoperative marking may reduce unnecessary invasion and potential complications; however, the frequency and characteristics of these lesions remain unclear. Therefore, we aimed to retrospectively assess the frequency and characteristics of pulmonary lesions for which a short hookwire was preoperatively placed but was deemed unnecessary during VATS.

## Materials and methods

This retrospective cohort study was approved by our institution's ethics committee (approval number: KEN2202-010), which waived the need for informed consent owing to its retrospective nature and utilization of existing medical information. An opt-out consent approach was used for retrospective data utilization. Written informed consent was obtained from each patient prior to preoperative short hookwire placement and VATS.

### Pulmonary lesions

Between June 2017 and November 2021, 176 preoperative short hookwire placements for pulmonary lesions were performed in 171 sessions at our institution. The decision of placement was based on one or more of the subsequent CT findings: a lesion with a diameter ≤ 10 mm; distance from pleural surface > 5 mm; and a lesion mostly consisting of ground-glass opacity [3].

### Short hookwire placement

An experienced radiologist or radiology trainee (resident or fellow), under the direct supervision of an experienced radiologist, performed the short hookwire placement under CT fluoroscopy guidance (Aquilion, Canon Medical Systems, Otawara, Japan) in an interventional radiology suite. The short hookwire-and-suture system comprises several components. The key component is the short hookwire itself, which has a diameter of 0.28 mm and a length of 10 mm. This short hookwire incorporates a 30-cm long 5–0 nylon monofilament suture securely affixed to the funnel located at the proximal end of the hookwire [2].

Following local anesthesia with lidocaine, a 21-gage introducer needle was advanced close to the lesion. The needle tip was optimally positioned, as verified via CT fluoroscopic imaging, and the pusher was fully deployed to release the short hookwire. The needle was then withdrawn cautiously. A chest CT scan, with a slice thickness of ≤ 5 mm, was subsequently obtained to confirm the accurate placement of the short hookwire and assess any complications. The external portion of the suture was trimmed and covered with sterile gauze before the patient was transferred to the surgical suite for VATS.

### VATS

Thoracoscopic surgery was performed under one-lung ventilation and general anesthesia. The surgical procedures included segmentectomy and wedge resection. The number and location of surgical incisions were selected by an experienced thoracic surgeon or surgical trainee (resident or fellow) under the direct supervision of an experienced surgeon to resect the lung lesion according to its clinical features. Lymph node excision was performed as required and resected tissues were extracted using a disposable wound retractor placed at the incision site.

### Evaluation of the need for short hookwire

The necessity of a short hookwire for resection was retrospectively assessed based on surgical records by a surgeon (H.U.) with 7 years of experience. The hookwire was regarded necessary when the following statements were found in the surgical records: “hookwire was used as a landmark” and/or “the lesion could not have been detected without hookwire.” A hookwire was considered unnecessary when the surgical records indicated that the lesion was identifiable (visible or palpable) without a hookwire. The surgical video recordings were reviewed and the necessity of a short hookwire was re-evaluated in the absence of relevant information in the surgical records.

### Data collection

Several patient, lesion, and procedural variables were evaluated to determine the need for a short hookwire. Patient variables included age, sex, presence of emphysema on preoperative chest CT images, pulmonary function test values (i.e., vital capacity [VC] and forced expiratory volume in 1 s [FEV1]), and history of previous ipsilateral lung resection. The tumor variables included size (long-axis diameter in mm), laterality (left lung or right lung), lobar location (upper lobe, middle lobe, or lower lobe), distance from the nearest pleura to the lesion (in mm), type (ground-glass or solid nodule), and pathologic diagnosis (benign, primary lung cancer, or metastatic lung cancer). The procedure variables were procedure time (min), adverse events (AEs) of short hookwire placement (i.e., pneumothorax and pulmonary hemorrhage), and time from hookwire placement to VATS (within or more than 1 h). All AEs were evaluated according to the Clavien–Dindo classification [4]. The procedure time was defined as the time between the initial CT examination and the CT examination performed immediately after hookwire placement. The presence of pulmonary adhesions to the chest wall during VATS was evaluated by a surgeon. In addition, the operator’s experience (surgical trainee or experienced surgeon) assessed based on surgical records.

### Statistical analysis

**Fig. 1:**
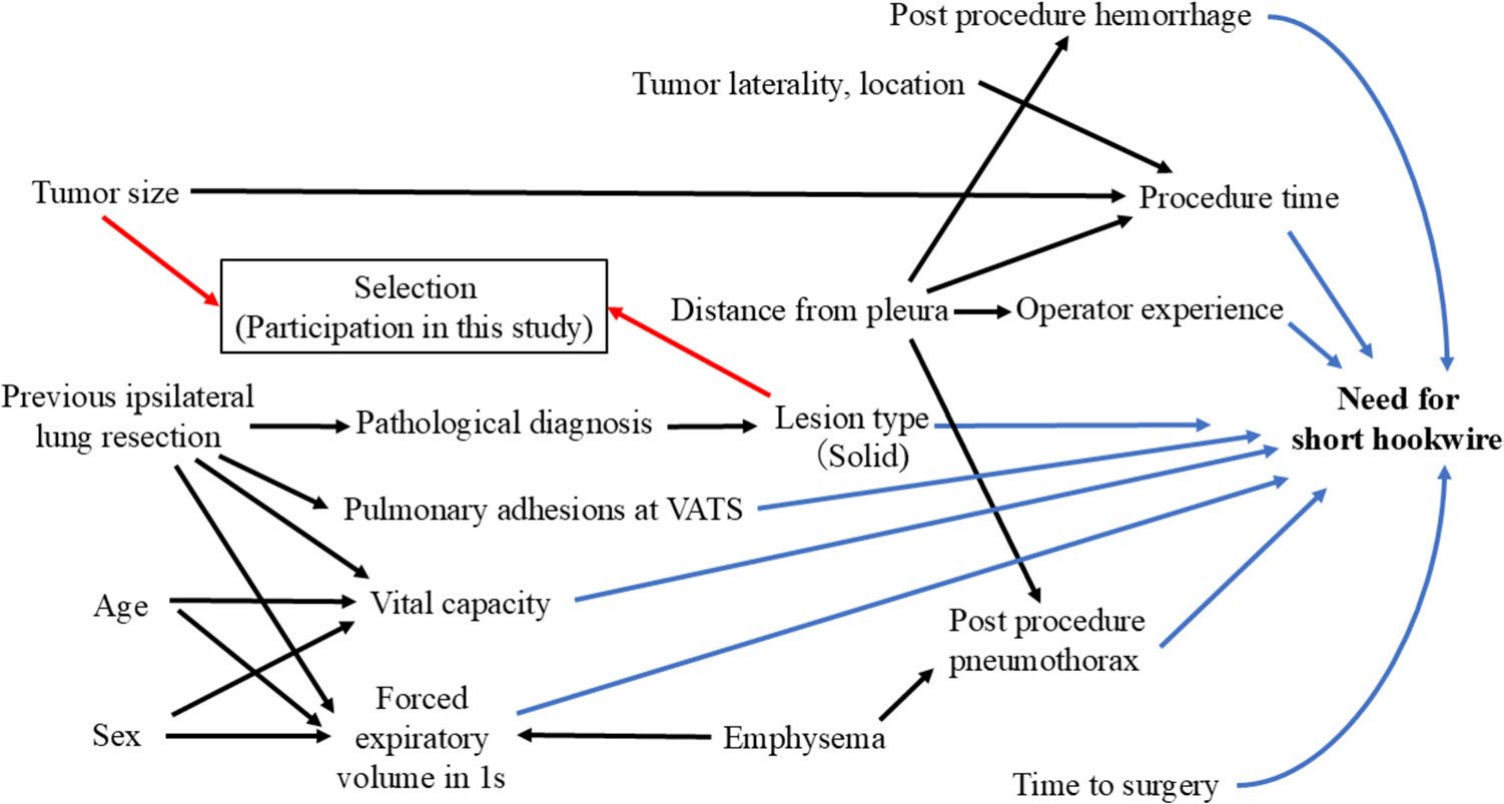
Directed acyclic graph representing the causal relationship between variables assumed by the authors

First, descriptive statistics were calculated. Continuous variables are presented as means and variances, while categorical variables are presented as frequencies and percentages. Next, univariate logistic regression analysis was performed to investigate the relationship between each variable and the outcome to calculate the odds ratios (OR) and 95% confidence intervals (CI). Covariates for the multivariate analysis were selected according to the directed acyclic graph (Fig. 1) and the modified disjunctive cause criterion for controlling potential confounders [5]. Receiver operating characteristic (ROC) curve analysis was used to evaluate the predictive ability of lesion size (long-axis diameter) and distance from the nearest pleura to the lesion for the need for hookwires. The optimum cut-off value for the ROC curve was determined based on Youden’s Index. Analyses were performed by an epidemiologist/biostatistician (T.M.) with 16 years of experience, using Stata 16.1/MP4 (Stata Corporation, College Station, TX, USA). Statistical significance was set at *P* < 0.05. Odds ratios (ORs) were calculated for the significant categorical variables. Missing values were not imputed or replaced and a complete case analysis was performed.

## Results

The patient characteristics, lesions, and procedures are shown in Table 1. In total, 165 patients (74 male and 91 female), with a mean age ± standard deviation (SD) of 63.6 ± 13.1 years (range 21–85 years), were included. Hookwire placement for two lesions was performed in the same session in five patients, while six patients underwent hookwire placement for two lesions in different sessions. All patients underwent preoperative pulmonary function tests. The mean VC was 3.0 ± 0.8 l (range 1.5–6.1 l) and FEV1 was 2.3 ± 0.7 l (range 0.9–5.7 l). Twenty-two patients previously underwent ipsilateral lung resection.

**Table 1:** Characteristics of the 165 patients, 176 lesions, and 176 placements

Variable	Value
Patient characteristics	
Age (years)	63.6 ± 13.1 (21–85)
Male sex	74 (44.8%)
Emphysema	25 (15.2%)
Vital capacity (l)	3.0 ± 0.8 (1.5–6.1)
Force expiratory volume in 1 s (l)	2.3 ± 0.7 (0.9–5.7)
Previous ipsilateral lung resection	22 (13.3%)
Tumor characteristics	
Size (mm)	7.9 ± 3.5 (2.3–22.4)
Right laterality	77 (43.7%)
Location	
Upper*	90 (51.1%)
Middle	9 (5.1%)
Lower	77 (43.8%)
Lesion type	
Ground-glass nodule*	105 (59.7%)
Solid nodule	71 (40.3%)
Distance from pleura (mm)	11.0 ± 9.2 (0–60.7)
Pathological diagnosis	
Benign*	13 (7.4%)
Primary lung cancer	108 (61.4%)
Metastatic lung cancer	55 (31.2%)
Procedure characteristics	
Procedure time(min)	16.8 ± 9.7 (8–98)
Post-procedure pneumothorax	68 (38.6%)
Post-procedure pulmonary hemorrhage	70 (40.0%)
From hookwire placement to VATS ≥ 1(hour)	101 (57.4%)
Operator experience	
Surgical trainee	120 (68.2%)
Experienced surgeon	56 (31.8%)
Pulmonary adhesions to the chest at VATS	55 (32.0%)

*Reference.VATS video-assisted thoracic surgery

Quantitative variables are expressed as means ± standard deviation; numbers in brackets are ranges

Of the 176 pulmonary lesions (105 ground-glass and 71 solid nodules), 77 were located in the right lung, 99 in the left lung, 90 in the upper lobe, nine in the middle lobe, and 77 in the lower lobe. The mean lesion size was 7.9 ± 3.5 mm (range 2.3–22.4 mm) and the mean distance between the lesion and the nearest pleura was 11.0 ± 9.2 mm (range 0–60.7 mm). Of the 176 lesions, 13 were pathologically diagnosed as benign and 163 were pathologically diagnosed as malignant (108 primary and 55 metastatic lung cancers).

The mean procedure time was 16.8 ± 9.7 min (range 8–98 min). AEs occurred in 124 of 176 procedures (70. 5%). Grade I AEs included pneumothorax in 68 procedures (38.6%) and pulmonary hemorrhage in 70 procedures (40.0%). CT after hookwire placement showed no obscuration of the lesion due to pulmonary hemorrhage. Grade IIIa AEs included pneumothorax with chest tube placement in two procedures, one of which was previously published as a case of lung laceration [6]. No AEs of grade IV or higher were observed.

**Fig. 2:**
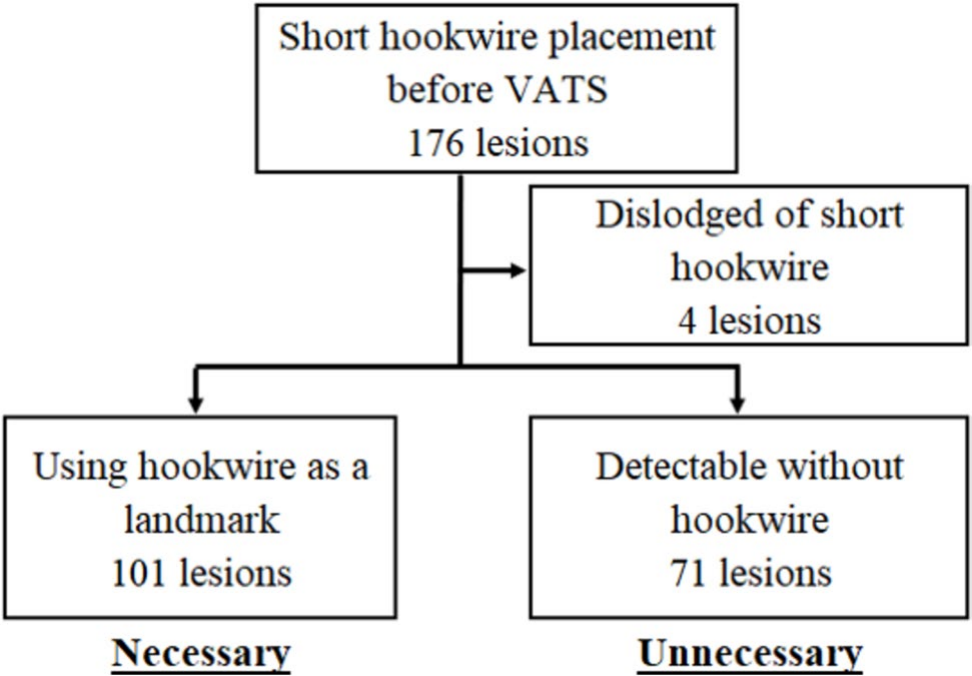
The need for short hookwire for VATS

VATS was initiated within 1 h of hookwire placement in 75 patients, whereas other patients waited for more than 1 h in their hospital rooms before undergoing VATS. The main operator of 120 (68.2%) VATS procedures was a surgical trainee under the direct supervision of an experienced surgeon. Adhesions between the lungs and chest wall were confirmed in 55 lesions by the surgeon during VATS. In addition, the hookwire was dislodged at the time of surgery in four lesions (2%). Among the remaining 172 lesions, thoracoscopic resection was performed using a hookwire as a landmark in 101 lesions (hookwire necessary), whereas 71 lesions were detectable without a hookwire (hookwire unnecessary) (Fig. 2).

**Fig.3:**
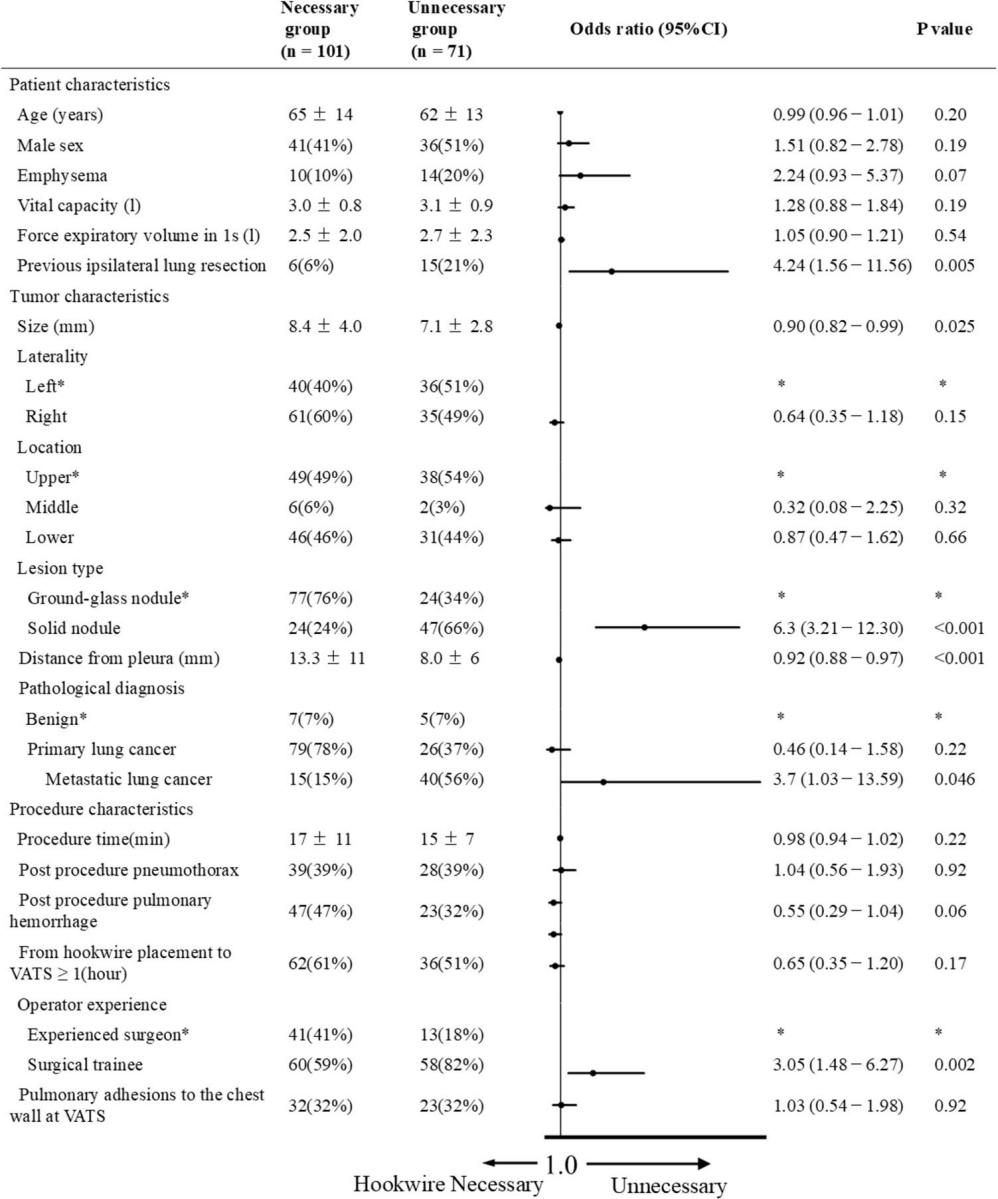
Forest plot of univariable analyses to determine the need for preoperative short hookwire. *Reference. *VATS* video-assisted thoracic surgery, *CI* confidence interval. Quantitative variables are expressed as means ± standard deviation. Qualitative variables are expressed as raw numbers

The variables for each group are summarized in Fig. 3. Previous ipsilateral lung resection significantly increased the odds of not needing a hook wire (OR 4.24; 95% CI 1.56–11.56; *P* = 0.005), whereas larger target lesions (mean, 8.4 vs. 7.1 mm; OR 0.90; 95% CI 0.82–0.99) and those located further from the pleura (mean, 13.3 vs. 8.0 mm; OR 0.92; 95% CI 0.88–0.97) were associated with an increased need for hookwires. In the comparison of lesion types, solid nodules (vs. ground-glass nodules; OR 6.3; 95% CI 3.21–12.30; P < 0.001) and metastatic lung cancer as a pathologic diagnosis (vs. benign; OR 3.7; 95% CI 1.03–13.59; P = 0.046) significantly increased the odds of not needing a hookwire. In addition, if the main operator of the VATS was a surgical trainee (vs. experienced surgeons; OR 3.05; 95% CI 1.48–6.27; *P* < 0.002), the odds of not needing a hookwire significantly increased.

Variables for multivariate analysis were selected from the patient, tumor and procedure characteristics based on a directed acyclic graph (DAG, Fig. 1) to adjust for potential confounders. Potential confounders requiring adjustment were selected using the DAG for each of the variables listed in Fig. 3, as shown in Table 2. Multivariate analyses were not performed for some variables (sex, age, emphysema, previous ipsilateral lung resection, tumor laterality and location, distance from the pleura, and time to surgery) because no potential confounders were identified. However, multivariate analyses were performed for the remaining factors, including risk factors and potential confounder(s), to determine the possibility of detecting the target lesion and revealed that solid nodules (OR 2.82; 95% CI 1.09–7.26; *P* = 0.032) and surgical trainee (OR 2.33; 95% CI 1.09–4.95; *P* = 0.029) significantly increased the odds of not needing a hookwire (Table 3).

**Table 2:** Confounding factors for each risk factor as determined by DAG

Risk factor	Potential confounder
Sex, age, emphysema, previous ipsilateral lung resection, tumor laterality and location, distance from pleura, time to surgery	None
Vital capacity	Sex, age, previous ipsilateral lung resection
Forced expiratory volume in 1 s	Sex, age, emphysema, previous ipsilateral lung resection
Lesion type	Tumor size, pathologic diagnosis
Tumor size	Lesion type
Procedure time	Tumor size, laterality, location, distance from pleura
Post-procedure pneumothorax	Emphysema, distance from pleura
Post-procedure hemorrhage, operator experience	Distance from pleura
Pathologic diagnosis, pulmonary adhesions at VATS	Previous ipsilateral lung resection

**Table 3:** Results of multivariable analyses to determine factors that make short hookwire placement unnecessary

Variables	Odds ratio	95%CI	*P* value
Patient characteristics			
Vital capacity (l)	1.03	0.61–1.75	0.92
Force expiratory volume in 1 s (l)	0.98	0.82–1.17	0.81
Tumor characteristics			
Size	0.99	0.890–1.11	0.92
Solid nodule (vs. ground-glass nodule)	2.82	1.09–7.26	0.032
Primary lung cancer (vs. benign)	0.46	0.13–1.58	0.22
Metastatic lung cancer (vs. benign)	3.38	0.91–12.51	0.07
Procedure characteristics			
Procedure time	0.97	0.93–1.02	0.20
Post-procedure pneumothorax	0.99	0.51–1.90	0.97
Post-procedure pulmonary hemorrhage	0.73	0.37–1.42	0.35
Surgical trainee (vs. experienced surgeons)	2.33	1.09–4.95	0.029
Pulmonary adhesions to the chest wall at VATS	0.67	0.32–1.41	0.29

*Reference. *VATS* Video-assisted thoracic surgery: *CI* confidence interval

**Fig.4:**
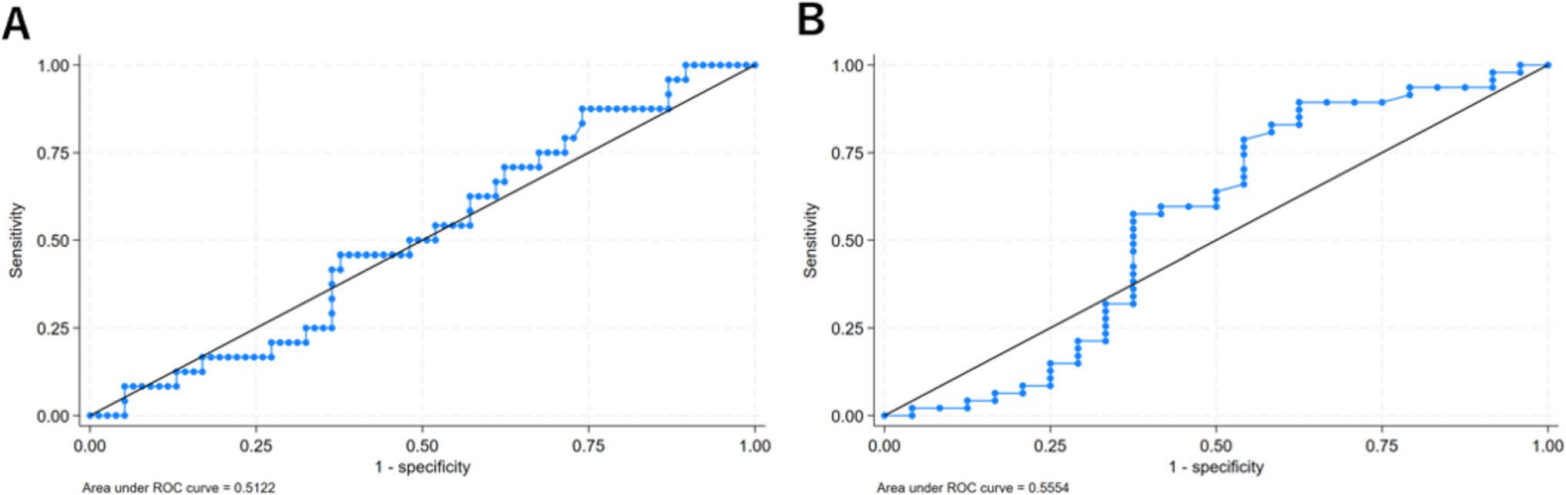
Receiver operating characteristics curves for lesion size (long-axis diameter) predicting the need for hook wires by lesion type (**A** ground-glass or **B** solid nodule)

**Fig.5:**
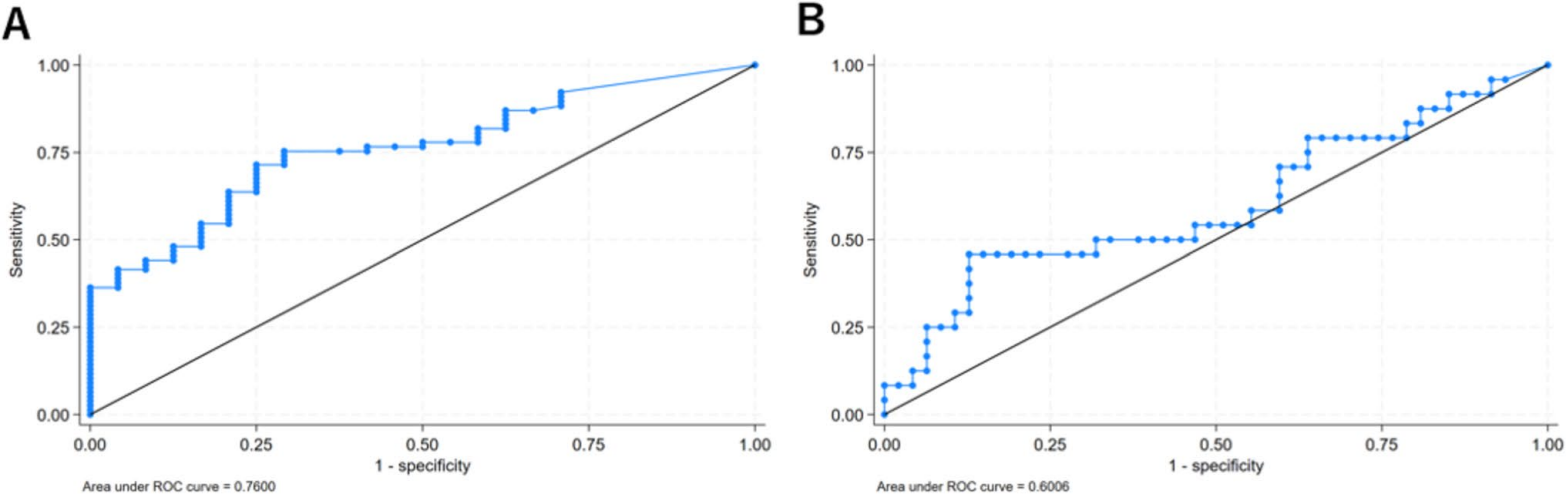
Receiver operating characteristics curves for distance from the nearest pleura to the lesion predicting the need for hook wires by lesion type (**A** ground-glass or **B** solid nodule)

Figure 4 shows the receiver operating characteristic curves (ROC) for lesion size (long-axis diameter) predicting the need for hookwires by lesion type (ground-glass or solid nodules). The area under the ROC curve (AUC) was 0.5122 for ground-glass nodules and 0.5554 for solid nodules. In addition, the minimum lesion size cut-off value predicting the need for a hookwire was 6.3 mm for ground-glass nodules and 3.8 mm for solid nodules. Figure 5 displays ROC curves for distance from the nearest pleura to the lesion predicting the need for hookwires by lesion type (ground-glass or solid nodules). The AUC was 0.7600 for ground-glass nodules and 0.6006 for solid nodules. In addition, minimum distance from the nearest pleura cut-off value predicting the need for a hookwire was 6.6 mm for ground-glass nodules and 14.1 mm for solid nodules. These results suggest that although solid nodules may be smaller and further away from the pleura than ground-glass nodules, they may not require hookwire placement.

## Discussion

In this study, we evaluated the need for short hookwire placement for preoperative marking according to our criteria and found that 58.7% of lesions (i.e., 101/172 lesions) required a short hookwire while 41.3% of lesions could be identified without a hookwire. In particular, some shallow solid lesions in patients with a history of ipsilateral lung resection may not require a short hookwire during VATS, even if they are small. Furthermore, our results revealed several cases in which surgical trainees with less years of experience determined that a hookwire was not necessary. Although our criteria have not changed in more than 15 years, minor modifications have been considered.

In 1995, Kanazawa et al. developed a short hookwire-and-suture system for preoperative localization [2], achieving high successful placement rates (range 97.6–99.6%) [3, 7–10]. If the target lung lesion cannot be identified, the strategy must be modified to ensure resection [3]. However, this modification results in increased invasiveness, including resection of a larger volume of lung parenchyma, increased patient wounding, and longer operative time, which should be avoided. In addition, the necessity of preoperative short hookwire placement is highly dependent on the institution and the individual surgeon’s skills (e.g., skill and experience with VATS). Therefore, thoracic surgeons prefer placing a short hookwire unless they are absolutely certain that it is unnecessary. Particularly, more experienced surgeons may be more inclined to prefer a hookwire placement based on past failed experiences.

Although complications of preoperative short hookwire placement are frequent [1], with some reports indicating that complications occur in more than 50% of the placements [7, 9], most of them are minor asymptomatic, and do not require treatment [1]. Common complications of this procedure are pneumothorax (incidence, 32.1–68.1%) and pulmonary hemorrhage (incidence, 8.9–41.6%). However, the procedure is not absolutely safe, with reports of very rare serious complications (e.g., as migration into the vessel [11], air embolization [12], pericardial pneumothorax [13]), and death [14]). Therefore, placing short hookwires for targets where marking is unnecessary is not in the patient’s best interest. Accordingly, our study highlights features of lesions that do not require preoperative short hookwire placement, which should aid clinicians in making the appropriate choice.

Several criteria for placement of the same short hookwire-and-suture system have been reported. Nakashima et al. reported the following criteria: a nodule with a maximum diameter ≤ 5 mm, maximum diameter of a nodule to the minimum distance between the visceral pleura and the inferior border of a nodule ≤ 0.5, and a nodule showing low density on CT imaging after chemotherapy [15]. Tamura et al. reported that targets were not detected when the target lesions were < 15 mm in diameter and the distance to the pleura was > 10 mm as the basis for the necessity of preoperative marking [16]. However, these were published in 2010 and may not necessarily be appropriate today, including the 2009 criteria [3]. In fact, approximately 40% of the resected lesions in this study could be detected without using a short hookwire.

Multivariate analysis in a previous study, wherein thoracic surgeons assessed the need for a short hookwire, revealed that the distance to the pleural surface (*P* = 0.0001) and ratio of the solid portion (*P* = 0.0104) were statistically significant [16]. Similarly, in our study, the need for a short hookwire was evaluated by the surgeons, and solid nodules, close proximity to the pleura, and previous ipsilateral lung resection were identified as factors that deemed this procedure unnecessary. In addition, ROC curve analysis was to evaluate the predictive ability of lesion size (long-axis diameter) and distance from the nearest pleura to the lesion for the need for hookwires in this study. The cut-off values for lesion size and distance from the nearest pleura to the lesion were given for ground-glass and solid nodules, respectively. A hookwire placement is generally required for ground-glass nodules based on conventional criteria (a diameter of ≤ 10 mm; distance from pleural surface > 5 mm); however, a hookwire may be unnecessary for solid nodules even if the diameter is smaller and the distance from the pleura is farther. Based on our findings, preoperative hookwire placement criteria should be reconsidered to incorporate individualized patient and lesion characteristics, such as lesion type, size, and distance from pleura, rather than adhering to a uniform approach for all cases.

Notably, in this study, experienced surgeons required more hook wires than trainees, which may be related to the fact that experienced surgeons were responsible for more difficult cases. Moreover, the surgical technique used in each case may have had minor differences, which could not be investigated in this study. Furthermore, the need for preoperative marking may not have been necessary in previous ipsilateral lung resection because: (1) the nature of the tumor is known from previous surgeries; therefore, predicting its tactile nature is easy, or (2) several metastatic lung tumors are present (owing to the large number of solid nodules). However, the exact reasons for these factors are unknown and require further verification.

Nonetheless, this retrospective study has some limitations. First, thoracic surgeon-related factors (e.g., years of experience) were not evaluated. More skilled individuals may need fewer preoperative markings, which may impact our results. Second, the time required to identify the tumor was not evaluated. One of the advantages of this system is that the intrathoracic suture from the lung surface can be used as a guide [1]. Thus, the use of hookwires may contribute to reduced operative time. Third, the same results may not be obtained when using other preoperative marking methods. Finally, the sample size may be insufficient, which may have reduced the detection power; therefore, future studies should consider increasing the sample size.

In conclusion, approximately 40% of the resected lesions can be detected without using a short hookwire. Some shallow solid lesions in patients with a history of ipsilateral lung resection may not require a hookwire during resection, even if they are smaller in size.
